# Olympic cycle periodicity in women’s long and triple jumping performance between 1996 and 2019

**DOI:** 10.1371/journal.pone.0286641

**Published:** 2023-06-08

**Authors:** Tim Taha, Jada Roach

**Affiliations:** Faculty of Kinesiology and Physical Education, University of Toronto, Toronto, Ontario, Canada; Universidad de Castilla-La Mancha, SPAIN

## Abstract

Performance variability is present in a series of competition results in athletics. Some of the variability is random and some can be attributed to factors such as the environment and changes in the level of physical, mental, and technical states of the athlete. Changes in the state of the athlete may be due to the competition schedule. It has been shown that there is periodicity in performance aligned with the seasonal competition schedule in athletics and with the Olympic cycle in pooled athletics data dating from 1896 to 2008. We investigated whether Olympic cycle periodicity was present in modern era long and triple jumping in elite men and women. Top 50 performances per year in the horizontal jumps in men and women from 1996 to 2019 were used. Each performance was normalized to the best result from the previous Olympic year. Two-way ANOVAs revealed significantly lower mean normalized performances in top ten women compared to top ten men (p < 0.001) in both jumps. In both jumps, ten top-performing women also showed decreases between the Olympic year mean normalized performances and the 1^st^ year following (Long Jump: p = 0.022, Triple Jump: p = 0.008). In triple jump, the decrease in performance was also found in the second year following the Olympics. Performances deciles ranked from 11^th^ to 50^th^ place showed a similar pattern in women’s triple jump but only for ranks 11 to 20 in the women’s long jump. The findings suggest that periodicity driven by the Olympic cycle exists in women’s long and triple jump at the elite level.

## Introduction

Variability has been shown to be present in athletics performances when a series of competition results are compared [1, 2]. As with any variability measure some of the range is due to measurement error [3] but the majority of variability is due to other factors such as changes in physiological, psychological and technical states between competitions [4]. Environmental factors may affect variability as well, with temperature and wind being two of the major contributors [5]. For example, higher temperatures may aid in sprint running events by lowering air resistance [6] but hinder performance in distance running events due to the high energy cost of heat removal by the human body [7]. In certain track and field events such as long and triple jumping, wind-aided distances are often excluded from records.

Variability in performance may also be intentional. Coaches and athletes periodize training programmes to achieve gains in certain abilities with a short-term loss in performance with the goal of increasing performance in the longer term [8]. If competitions are attempted during periods of higher intensity or higher duration training, performances will be lower than later performances as the body transmutes the physiological changes into increased sport performance. The variability in performance may occur within a period of weeks during a single mesocycle or within a longer period of time such as a single season. Intentional performance variability may also be caused by weight manipulation [9], illegal doping or therapeutic use exemptions (TUE) for performance enhancing drugs [10]. Changes in performance during a season have been shown to be due to factors such as the timing of major competitions or championships [11]. Cultural peaks in performance in athletics occur during the mean 34^th^ week of the year which corresponds with the timing of major competitions such as the Olympic games, World Championships or European Championships [11]. In addition, a secondary season peak occurs earlier in the year corresponding to national championships or competitions that allow qualification for major events [11]. Periodicity of performance has been noted in relation to the Olympic cycle using combined performance data from 36 women’s and men’s track and field events from 1896 to 2008 [12]. In that data, there was a 0.99% increase in Olympic year performance followed by a 0.32% decrease for the post-Olympic year. The remaining two years in the Olympic cycle showed increases of 0.48% and 0.37%. With changes in training methodology and the introduction of the World Championships in the 1970s, it is possible that those patterns of periodicity may have changed in the modern World Championship era.

Differences in performances between men and women in Athletics has been extensively examined [13]. Both longitudinal evaluations [14] of gender-based performance differences over long time frames and cross-sectional differences in shorter time frames [15] have been conducted for events in Athletics. Long term studies have indicated that gender differences have been persistent in both differences in absolute performance (e.g., running times, distances jumped) and modeled power production [14]. In the horizontal jumps, differences between men’s and women’s performances have been found to range between 17 and 20% in the long jump and 15 and 18% in the triple jump in the years from 1983 to 2015 depending on the curve fitting approach used [15]. These approaches have not looked at the differences in relative performance of athletes compared to top performances. For example, if the mean of the top 10 performances of a given year is compared to a benchmark such as top Olympic year performance, how do men and women compare? These differences in normalized performances can indicate the depth of competition. A lower normalized mean performance would indicate that there is a greater difference between the top performance and the other performances.

Both the long and triple jumps involve an approach run of up to about 40m to a take-off board which indicates the line from the distance is the jump is measured. In the long jump, the athlete leaves the ground with a single foot take-off for the flight phase and lands in a sandpit. The mark made by the athlete in the sandpit upon landing that is closest to the take-off board is used to measure the jump distance from the take-off board. The triple jump has two ground contacts and three flight phases following the last ground contact at the take-off board. The first ground contact is the same foot as the take-off foot and is considered a hop, the second is then made with the opposite foot and is called a step or skip. The final flight phase is into the sandpit and is like the long jump flight phase. Measurement in the triple jump is the same as the long jump back to the take-off board. Performance in the long [16] and triple jumps [17] has been shown to be strongly linked to horizontal running speed prior to the last contact at the take-off and the ability to exchange that velocity with vertical velocity [18].

In athletics, the quadrennial calendar is dominated by the Olympics. In addition, beginning in 1991, the International Amateur Athletic Federation (IAAF, now World Athletics) added biennial athletics world championships that are held in the summers following and preceding the Olympic Games [19]. We chose the horizontal jumps as one author currently competes in both jumps and the other coaches sprinters who compete in jumps. We have observed decreases in jumping competition performance between Olympic years anecdotally and wished to see whether this pattern emerged when analysed empirically and statistically. In the current study, we hypothesized that there is a cultural or secular effect on horizontal jump performance due to the timing of major competitions during the Olympic quadrennial. Our primary aim, therefore, was to observe the changes in horizontal jump performance relative to performances in the preceding Olympic years to see whether they decrease, increase, or remain constant during the 4-year Olympic cycle. We also aimed to see whether the changes in horizontal jump performance relative to preceding Olympic year performances occurred in both men and women.

## Methods

### Data

The top fifty outdoor long jump and triple jump distances for each year between 1996 and 2019 were accessed from the World Athletics website [20] for both men and women. Each of the distances were coded depending on the year within the four-year Olympic cycle they fell. The Olympic year (1996, for example) was coded as year zero and the first, second and third year following the Olympic year were coded 1, 2 or 3, respectively.

### Design and procedure

The study was a quasi-longitudinal design. Multiple years (e.g., 1997, 2001, 2005) were recoded as years within the Olympic cycle. This allowed mean performances to be tracked over the 4-year Olympic cycle. Each individual performance was normalized to the farthest distance of the Olympic year of that particular Olympic cycle for event and sex. For example, a women’s long jump performance in 1999 would be normalized to the best women’s long jump distance of the Olympic year 1996 and coded as Olympic cycle year 3. The data of each event by sex was then sliced into five groups consisting of the first through 10^th^ performance by year, the 11^th^ through 20^th^ performances and so on up to the 41^st^ to 50^th^ performance. This allowed us to see whether top performances showed patterns that differed from lower performances in the Olympic quadrennial cycle.

### Statistical analysis

All statistical analysis were completed using R [21] with a significance level set at α = 0.05. The normalized mean performances were checked for normality using a Shapiro-Wilkes test procedure. All assumptions for normality were met. For each of the jumps, separate two-way ANOVAs were run for each of decile slices resulting in a total of ten two-way ANOVAs. For example, long jump with the 21st to 30th ranked normalized data was one of the ANOVAs run. In each of the ANOVAs, sex (Men, Women) and Olympic cycle year (0, 1, 2, 3) were used as main effects and sex by Olympic cycle year was an interaction effect. Effect sizes were calculated using Cohen’s f statistic [22]. An f statistic of 0.10, 0.25 and 0.40 indicates small, medium, and large effect sizes respectively. Tukey post-hoc tests were used to assess the differences between means.

The study was approved by the University of Toronto Faculty of Kinesiology and Physical Education ethics review committee as conforming to all relevant ethical policies. As all data was publicly available, no consent was required by the ethics review committee.

## Results

**Fig 1:**
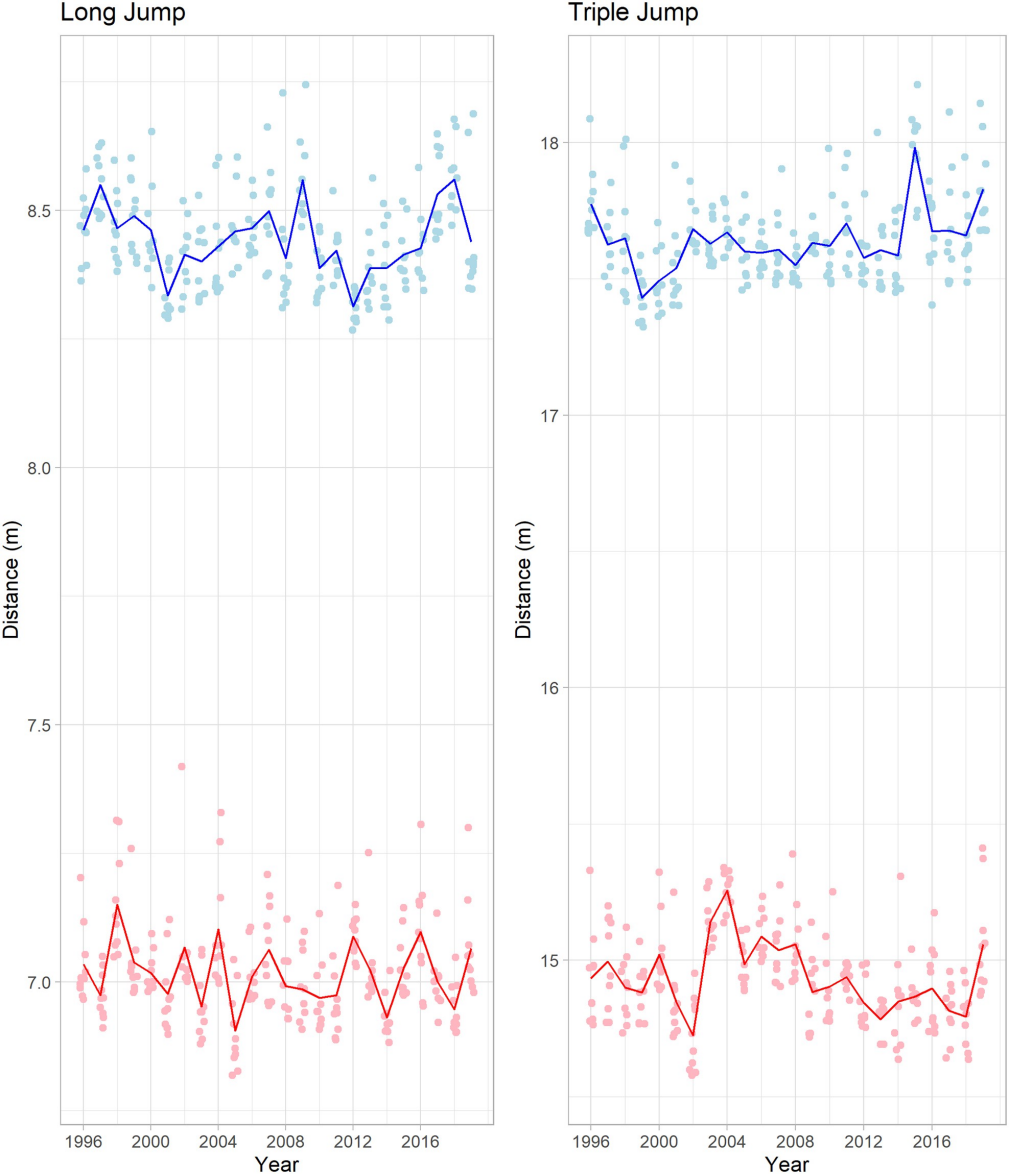
Top ten performances by year in long and triple jump in men and women. Points are individual performances, and the lines are the mean performance of the top ten. The blue (top) line are men, and the red (bottom) line are women.

In total, 4800 outdoor jump distances were collected. Of these, 2400 were long jump distances and 2400 were triple jump distances. During the period studied (1992 to 2019), no world records were set in both men’s and women’s long jump performances. In contrast, outdoor world records were set on four occasions in the women’s triple jump and on three occasions in the men’s triple jump. The progression of top 10 long and triple jump performances is shown in Fig 1.

### Analysis 1: Changes in top ten long jump performances by Olympic cycle year and sex

**Fig 2:**
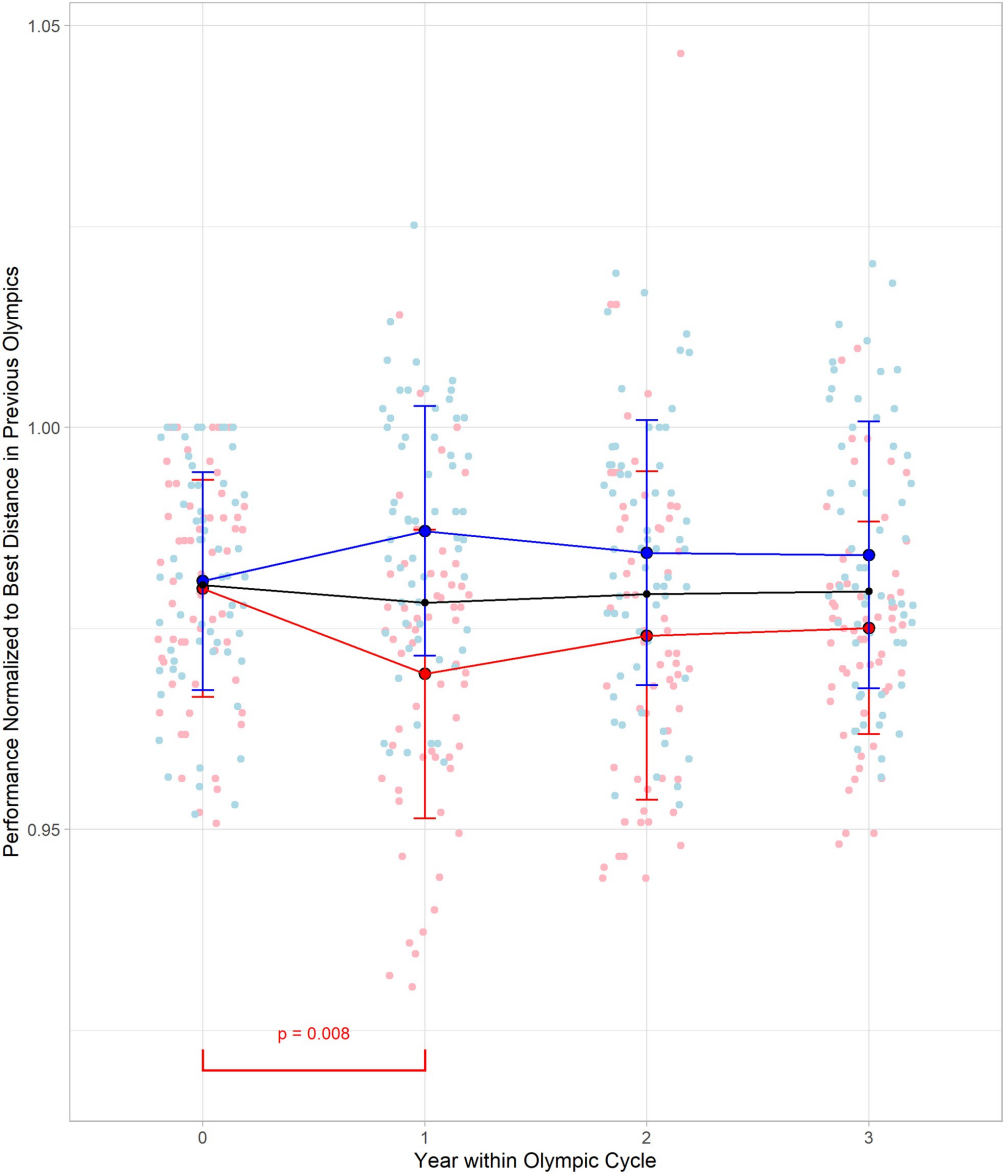
Change in mean normalized top ten performances in men and women’s long jump by Olympic cycle year. Points are individual performances and the lines are the mean normalized performance of the top ten. The blue (top) line are men, and the red (bottom) line are women. The black (middle) line is the group values.

In the top 10 men’s and women’s long jump performances there was no main effect for Olympic cycle (F = 0.386, p = 0.763), but there was a main effect for sex (F = 42.132, p < 0.001, Cohen’s *f* = 0.30). An interaction between sex and Olympic cycle year (F = 5.527, p < 0.001, Cohen’s *f* = 0.19) was also found. A Tukey post-hoc test reveled significant differences between Olympic cycle year 0 and 1 (p = 0.008) normalized jump performances in females (Fig 2). No other differences were found within either women’s or men’s normalized jump performance when compared between Olympic cycle year. Differences were also found between men’s and women’s mean normalized performances in Olympic cycle years (Table 1).

**Table 1:** Interactions between mean normalized men’s and women’s long jump performances for top 10 rankings. Shown as difference of Men—Women, p-value.

Sex, Olympic Cycle Year	Men, year 0	Men, year 1	Men, year 2	Men, year 3
Women, year 0	NS	NS	NS	NS
Women, year 1	0.01, p = 0.002	0.02, p < 0.001	0.02, p < 0.001	0.01, p < 0.001
Women, year 2	NS	0.01, p < 0.001	0.01, p = 0.011	0.01, p = 0.033
Women, year 3	NS	0.01, p < 0.001	0.01, p = 0.031	0.01, p = 0.044

NS = Not significant

### Analysis 2: Changes in top ten triple jump performances by Olympic cycle year and sex

**Fig 3:**
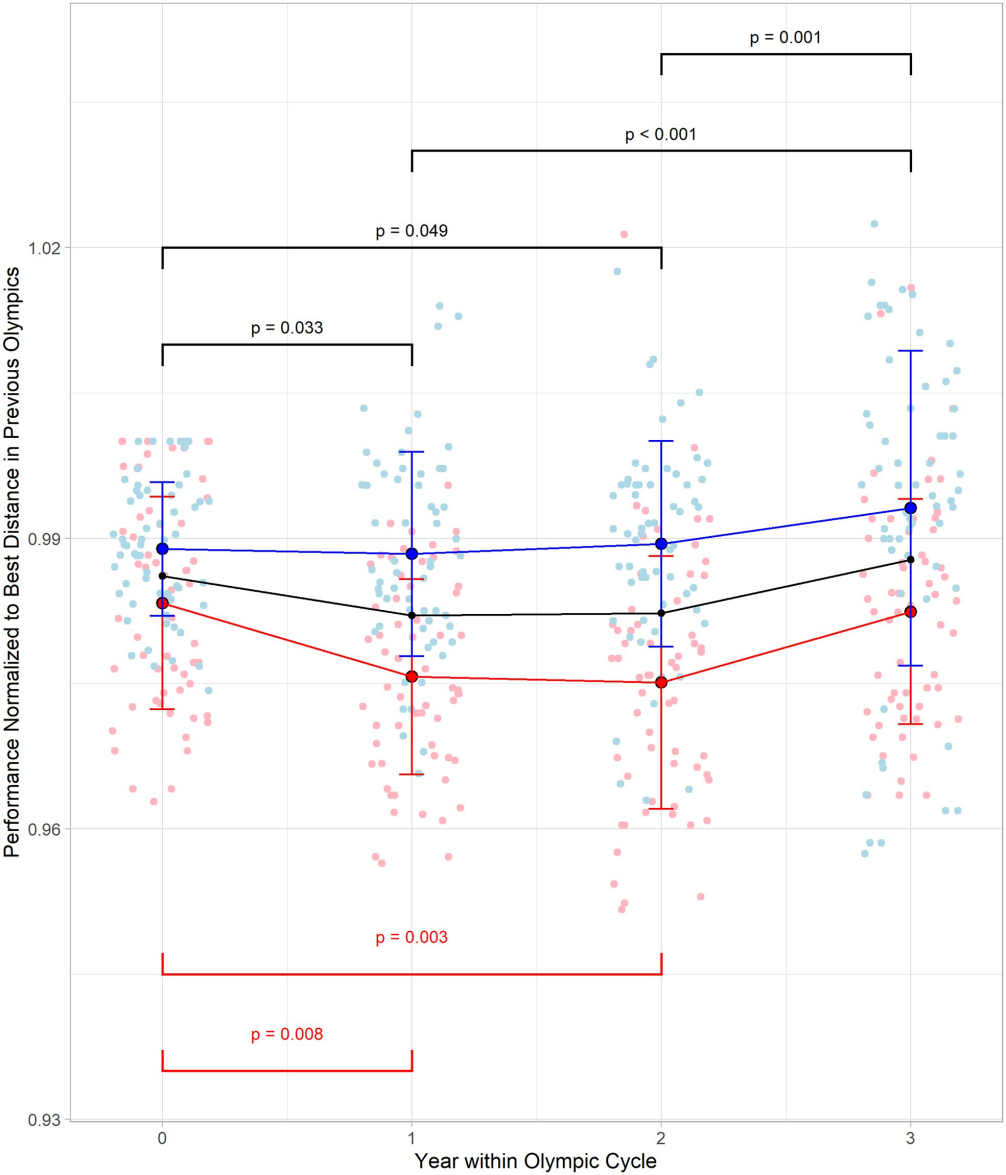
Change in mean normalized top ten performances in men and women’s triple jump by Olympic cycle year. Points are individual performances and the lines are the mean normalized performance of the top ten. The blue (top) line are men, and the red (bottom) line are women. The black (middle) line is the group values. Significant differences are shown with the red bars (lower) representing the differences in women’s mean normalized performances and the black bars (upper) representing the differences in the group mean normalized performances.

For the top 10 men’s and women’s triple jump performances there was a main effect of Olympic cycle year (F = 7.297, p < 0.001, Cohen’s *f* = 0.21). There was also a main effect of sex (F = 105.514, p < 0.001, Cohen’s *f* = 0.47) and a significant interaction between sex and Olympic cycle year (F = 3.234, p = 0.022, Cohen’s *f* = 0.14). Post-hoc testing revealed that the main effect differences were between Olympic cycle year zero and one (p = 0.033) and years zero and two (p = 0.049) as well as significant differences between years one and three (p < 0.001) and years two and three (p = 0.001) as shown in Fig 3. Interactions between sex and Olympic cycle year were found to be significant (p = 0.021) with a Tukey post-hoc analysis indicating significant decreases in normalized performances in females between Olympic cycle years zero and one (p = 0.008) and years zero and two (p = 0.003). Additionally, the Tukey post-hoc test indicated differences between men’s and women’s performances both within and between Olympic cycle years (Table 2).

**Table 2:** Interactions between mean normalized men’s and women’s triple jump performances for top 10 rankings. Shown as difference of Men—Women, p-value.

Sex, Olympic Cycle Year	Men, year 0	Men, year 1	Men, year 2	Men, year 3
Women, year 0	NS	NS	NS	0.01, p < 0.001
Women, year 1	0.01, p < 0.001	0.01, p < 0.001	0.01, p < 0.001	0.02, p < 0.001
Women, year 2	0.01, p < 0.001	0.01, p < 0.001	0.01, p < 0.001	0.02, p < 0.001
Women, year 3	0.01, p = 0.045	NS	0.01, p < 0.021	0.01, p < 0.001

NS = Not significant

### Analysis 3: Changes in long jump performances by Olympic cycle year and sex in places 11 to 50

The third analysis looked at the changes in long jump performances between Olympic cycle year and sex in performances that were ranked 11^th^ to 50^th^. The performances were separated into decile groups of rankings from 11^th^ to 20^th^ rank, 21^st^ to 30^th^ rank, 31^st^ to 40^th^ rank and 41^st^ to 50^th^ rank. A different ANOVA was used for each decile. In the long jump, there were main effects for sex in all four deciles (F < 85, p <0.001 for all four deciles, Cohen’s *f* = 0.43 to 0.49). Interactions were only found for the 11^th^ to 20^th^ rank and 21st to 30^th^ deciles (F = 6.466, p < 0.001, Cohen’s *f* = 0.20 and F = 3.062, p = 0.028, Cohen’s *f* = 0.15, respectively). Tukey post hoc testing revealed that within women ranked 11^th^ to 20^th^, there was a significant decrease in performance from Olympic cycle year zero to year one (p < 0.001). Further significant differences were found between men’s and women’s performances both within and between Olympic cycles years for ranks 11^th^ to 20^th^ and 21^st^ to 30^th^ (Tables 3 and 4).

**Table 3:** Interactions between mean normalized men’s and women’s long jump performances for rankings 11 to 30. Shown as difference of Men—Women, p-value for each decile ranking.

Ranking	Sex, Olympic Cycle Year	Men, year 0	Men, year 1	Men, year 2	Men, year 3
11 to 21	Women, year 0	NS	NS	NS	NS
	Women, year 1	0.01, p < 0.001	0.02, p < 0.001	0.02, p < 0.001	0.02, p < 0.001
	Women, year 2	0.01, p = 0.015	0.01, p < 0.001	0.01, p < 0.001	0.01, p < 0.001
	Women, year 3	0.01, p = 0.005	0.01, p < 0.001	0.01, p < 0.001	0.01, p < 0.001
21 to 30	Women, year 0	0.01, p = 0.020	0.01, p < 0.001	0.01, p = 0.002	0.01, p = 0.011
	Women, year 1	0.02, p < 0.001	0.02, p < 0.001	0.02, p < 0.001	0.02, p < 0.001
	Women, year 2	0.01, p < 0.001	0.01, p < 0.001	0.01, p < 0.001	0.01, p < 0.001
	Women, year 3	0.01, p < 0.001	0.01, p < 0.001	0.01, p < 0.001	0.01, p < 0.001

**Table 4:** Main effects of Olympic cycle year on mean normalized men’s and women’s long jump performance.

Rankings	Olympic Cycle Year Comparison	Difference	p value
1 to 10	1–0	-0.004	0.033
	2–0	-0.004	0.050
	3–1	0.005	<0.001
	3–2	0.005	0.001
11 to 20	1–0	-0.004	0.018
	2–0	-0.004	0.011
	3–1	0.005	<0.001
	3–2	0.005	<0.001
21 to 30	2–0	- 0.003	0.028
	3–1	0.004	0.012
	3–2	0.004	0.001
31 to 40	3–1	0.003	0.015
	3–2	0.003	0.015
41 to 50	3–1	0.003	0.044

### Analysis 4: Changes in triple jump performances by Olympic cycle year and sex in places 11 to 50

In the triple jump performances ranked 11th to 50th, all four deciles (11th to 20th, 21st to 30th, 31st to 40th and 41st to 50th ranks) had main effects for Olympic cycle year (11th to 20th: F = 8.909, p < 0.001, Cohen’s *f* = 0.24; 21st to 30th: F = 6.128, Cohen’s *f* = 0.20, p < 0.001, 31st to 40th; F = 4.370, p = 0.005, Cohen’s *f* = 0.17; 41st to 50th: F = 2.990, p = 0.031, Cohen’s *f* = 0.14) and sex (all deciles F > 235, p < 0.001, Cohen’s *f* = 0.71 to 0.89). All four deciles also had significant interactions between Olympic cycle year and sex (11^th^ to 20^th^: F = 5.570, p < 0.001, Cohen’s *f* = 0.19; 21^st^ to 30^th^: F = 6.128, p < 0.001, Cohen’s *f* = 0.19; 31^st^ to 40^th^: F = 4.370, p = 0.004, Cohen’s *f* = 0.17; 41^st^ to 50^th^: F = 2.990, p = 0.017, Cohen’s *f* = 0.15). Tukey post-hoc tests revealed significant differences in normalized performances between Olympic cycle years in all four deciles between 11^th^ and 50^th^ rankings (Table 5). While there were no significant differences in normalized triple jump performances between Olympic cycle years in men, women had several significant differences in those four deciles (Table 6). Further significant differences were found in the interactions between men and women and Olympic cycle years (Table 7).

**Table 5:** Main effects of Olympic cycle year on mean normalized men’s and women’s triple jump performance.

Rankings	Olympic Cycle Year Comparison	Difference	p value
1 to 10	1–0	-0.004	0.033
	2–0	-0.004	0.050
	3–1	0.005	<0.001
	3–2	0.005	0.001
11 to 20	1–0	-0.004	0.018
	2–0	-0.004	0.011
	3–1	0.005	<0.001
	3–2	0.005	<0.001
21 to 30	2–0	- 0.003	0.028
	3–1	0.004	0.012
	3–2	0.004	0.001
31 to 40	3–1	0.003	0.015
	3–2	0.003	0.015
41 to 50	3–1	0.003	0.044

**Table 6:** Interaction effects of Olympic cycle year on mean normalized triple jump performance in women.

Rankings	Olympic Cycle Year Comparison	Difference	p value
1 to 10	1–0	-0.008	0.008
	2–0	-0.008	0.003
11 to 20	1–0	-0.007	0.002
	2–0	-0.008	<0.001
	3–1	0.007	<0.001
	3–2	0.008	<0.001
21 to 30	1–0	- 0.005	0.029
	2–0	- 0.007	<0.001
	3–1	0.006	0.003
	3–2	0.008	<0.001
31 to 40	2–0	-0.005	0.041
	3–1	0.006	0.005
	3–2	0.007	<0.001
41 to 50	3–1	0.005	0.022
	3–2	0.006	0.002

**Table 7:** Interactions between mean normalized men’s and women’s triple jump performances for rankings 11 to 50. Shown as difference of Men—Women, p-value for each decile ranking.

Ranking	Sex, Olympic Cycle Year	Men, year 0	Men, year 1	Men, year 2	Men, year 3
11 to 21	Women, year 0	0.01, p < 0.001	0.01, p < 0.001	0.01, p < 0.001	0.01, p < 0.001
	Women, year 1	0.02, p < 0.001	0.02, p < 0.001	0.02, p < 0.001	0.02, p < 0.001
	Women, year 2	0.02, p < 0.001	0.02, p < 0.001	0.02, p < 0.001	0.02, p < 0.001
	Women, year 3	0.01, p < 0.001	0.01, p < 0.001	0.01, p < 0.001	0.01, p < 0.001
21 to 30	Women, year 0	0.01, p < 0.001	0.01, p < 0.001	0.01, p < 0.001	0.01, p < 0.001
	Women, year 1	0.02, p < 0.001	0.02, p < 0.001	0.02, p < 0.001	0.02, p < 0.001
	Women, year 2	0.02, p < 0.001	0.02, p < 0.001	0.02, p < 0.001	0.02, p < 0.001
	Women, year 3	0.01, p < 0.001	0.01, p < 0.001	0.01, p < 0.001	0.01, p < 0.001
31 to 40	Women, year 0	0.01, p < 0.001	0.01, p < 0.001	0.01, p < 0.001	0.01, p < 0.001
	Women, year 1	0.02, p < 0.001	0.02, p < 0.001	0.02, p < 0.001	0.02, p < 0.001
	Women, year 2	0.02, p < 0.001	0.02, p < 0.001	0.02, p < 0.001	0.02, p < 0.001
	Women, year 3	0.01, p < 0.001	0.01, p < 0.001	0.01, p < 0.001	0.01, p < 0.001
41 to 50	Women, year 0	0.01, p < 0.001	0.01, p < 0.001	0.02, p < 0.001	0.01, p < 0.001
	Women, year 1	0.02, p < 0.001	0.02, p < 0.001	0.02, p < 0.001	0.02, p < 0.001
	Women, year 2	0.02, p < 0.001	0.02, p < 0.001	0.02, p < 0.001	0.02, p < 0.001
	Women, year 3	0.01, p < 0.001	0.01, p < 0.001	0.01, p < 0.001	0.01, p < 0.001

## Discussion

Significant patterns emerged from Olympic quadrennial normalized horizontal jump performance data in the current study. In top ten performances, there were significant decreases in women’s mean performances between the Olympic year and the first post-Olympic year in both long and triple jump. In triple jump, a significant decrease in mean performance was also found between the Olympic year and the second post Olympic year for the entire group. However, neither of these two findings were reflected in the men’s mean performances but only in the women’s mean performances suggesting that the changes were driven by the women. Performance deciles ranked from 11^th^ to 50^th^ place showed a similar pattern in women’s triple jump but only for ranks 11 to 20 in the women’s long jump. Differences in mean normalized performance were also found between men and women with women having lower mean normalized performances across multiple years in the Olympic quadrennial in both long and triple jump across all rank deciles.

The transient decrease in women’s normalized performances during the first non-Olympic year in long and triple jump is a unique finding. There are few studies that look at changes in performances over an Olympic quadrennial. Rüst et al., looked at top ten triathlon performances in men and women between 2009 and 2012. They found that there was an overall increase in race times during that period [23] which indicates a decrease in performance during that quadrennial. A case study on a male and a female Canadian mogul skier found increased performances in various vertical jump heights over the quadrennial leading to the 2010 Winter Olympic Games [24]. The largest increase in jump height was found between the first and second year of the quadrennial. In broad jump, on the other hand, performance decreased over the quadrennial for both skiers [24]. The vertical jump findings in that study correspond to the findings in the current study but do not give insight to whether the performances in changed in the first year of the quadrennial compared to the preceding Olympic year performances.

The drop in mean top ten normalized performance in the year after the Olympics in both women’s long and triple jump is intriguing. Berthelot et al., studied the Olympic periodicity of the top performance of all Olympic athletics events between 1891 and 2008 [12]. They found that the mean improvement in performance varied by year in the Olympic cycle with the Olympic year leading to a 0.99% improvement in performance. The mean improvement was -0.32% in the year following the Olympics and 0.48% and 0.37% for years 2 and 3 following the Olympics. Berthelot’s findings are similar in direction to our findings in the women’s normalized mean performances in the top 2 deciles in long jump and top 5 deciles in triple jump. There are many possible reasons that may be responsible for our findings. Like the study by Haïda et al., which suggested that in seasonal peaks in performance were due to cultural reasons such as the timing of championships [25], it is possible that athletes and coaches use a similar methodology over the Olympic quadrennial with planned performance peaks at the Olympics. This conclusion suggests, however, that the World Championships in the season immediately following the Olympic games are not considered very important by athletes or coaches. In addition, it would be expected to observe the same changes in men’s performances which is not found in the current study’s results. Another possibility is the use of a multi-year periodization scheme with more lower intensity work during the post-Olympic cycle and then a building of intensity and volume of training through to the next Olympic games [8]. Multi-year periodization schemes have been proposed but there is scant empirical literature that demonstrates their use. For example, a case study looking at a Nordic combined athlete found year over year performance improvements over an Olympic quadrennial [26]. Top 150 place swimmers have also shown year over year improvements in performance from 2004 to 2008 [27]. In that swimming study, the athletes tracked had performances over all five seasons from 2004 to 2008, so those who stopped competing in 2005 would be excluded. The current study includes athletes who may have competed at an Olympics and then retired or stepped away from competition for one or more years. What we observed in the current study amongst women may reflect top performing athletes dropping out of competition following an Olympic Games. It has been found that a significant proportion of top performing women drop out of competition for family reasons which may include childcare or pregnancy [28]. Dropout for these reasons in women was found to be double that of men [28]. In the case of pregnancy, this may be a cessation of competition and training for a single season which could account for the skew in our post-Olympic year data if women athletes want to ensure the longest possible period of planned uninterrupted training and competition to the next Olympics.

The drop in mean normalized performance from the Olympic to the first post-Olympic year was present in women’s long and triple jump in the top ten decile and the 11^th^ to 20^th^ ranks decile. The drop in performance persisted in women’s triple jump performances in all five deciles. The findings from the long jump could be due to top performers following a training and competition schedule that is focused on Olympic results and lower ranked jumpers working to qualify for the Olympics. The other possibility may be that top jumpers are more likely to retire or not compete in the year following the Olympics. The difference between the two events may be due to the difference in the number of competitions that athletes take part in during a season. Triple jumping has very high loading on the body, approaching 16 times body weight on a single leg [29]. This high loading means that the number of full approach triple jumps that are completed in a season is limited [30]. It is possible that triple jump athletes chose to limit their competitions to major events and take longer periods of time from competition. Differing numbers of competitions between athletes may also affect the year-to-year variability in normalized mean performances. The differences in number of competitions may be due to the value that coaches and athletes place on competitions as training and whether athletes choose to compete in other events. For example, if competitions are seen as detrimental to training induced performance improvements, then fewer competitions may be entered.

Lower normalized mean performances were found for women when compared to men for both the long and triple jump at the same Olympic cycle years. It is important to note that we used the top performance from the Olympic year to normalize performances. What the results suggest is that the mean relative performance for the women is significantly lower than the men in both jumps and across all five deciles of performance ranks. While morphological and physiological differences would account for any disparity in absolute performances, those factors would not impact relative performances. One of the potential explanations for the difference may be due to limits on access for women in sports around the world. Increased world population has been associated with improved performance in humans, racing dogs and racehorses [31]. However, as the recent increases in world population has been much higher in regions with low women’s sport participation [32, 33], the relationship between performance and population has become disconnected in women. This disconnect can potentially result in decreased depth in women’s performances when compared to men.

Findings in the current study add to previous work that found that periodicity in performance may be due to cultural factors such as major competitions [11]. The periodicity in performance both in terms of the change in performance [12], and in terms of relative performance has implications that will make measuring changes in performance due to major disruptions such as COVID-19 difficult. Performance expectations at specific events such as the 2020 Tokyo Olympics (held in 2021) need to consider these different periodicities.

The findings in the current study also contribute to the understanding of the slowing of World Record progression. Berthelot et al., have found that 64% of Athletics events have stopped progressing between 1992 and 2008 [12]. This effect was stronger in women’s events with 78% stopping progression between 1992 and 2008. If the post-Olympic year decrease in mean performance is due to changes in training structure such as higher duration, lower intensity work as is often recommended in the first year of a multiyear periodization scheme [8], then it is possible that physical performance progression in high-intensity events such as the horizontal jumps is thwarted [34]. For example, the lack of high-intensity specific training may lead to decreases in contractile proteins [35] which, in turn, may hinder the force production necessary for high performance in jumping.

The major limitation to the current study is the result of using a population level approach to study the changes in performance within the Olympic quadrennial. Tracking individual athletes would help answer some of the questions arising from our findings. However, simply using performance data may not be able to explain the decrease in performances in the first post Olympic year. Qualitative research (interviews with athletes and coaches) would certainly yield useful information about behaviours that influence performance in the first post Olympic year. Obviously, as we have only studied the horizontal jumps, we are limited from making inferences about other events. Other events may have different incentives to perform, lower loading patterns which may make more competitions possible and different competition scheduling outside of the major events. For example, in marathon running certain events such as the Berlin marathon are popular amongst top performers due to the increased likelihood of setting personal best times [36].

## Conclusion

In the horizontal jumps, women’s mean normalized top ten mean performances decreased from the previous Olympic year’s performance in the first post Olympic year. In addition, in the women’s triple jump, a decrease was also found for top ten performances in the second post Olympic year when compared to the Olympic year. The decreases in performance in the first post Olympic year was found for all five deciles in the top fifty women’s triple jump performances and in the two deciles in the top twenty long jump performances. The findings suggest that periodicity exists in elite horizontal jump performances that appear to be driven by the Olympic quadrennial.
